# Seroprevalence of SARS-CoV-2–Specific Antibodies, Faroe Islands

**DOI:** 10.3201/eid2611.202736

**Published:** 2020-11

**Authors:** Maria Skaalum Petersen, Marin Strøm, Debes Hammershaimb Christiansen, Jógvan Páll Fjallsbak, Eina Hansen Eliasen, Malan Johansen, Anna Sofía Veyhe, Marnar Fríðheim Kristiansen, Shahin Gaini, Lars Fodgaard Møller, Bjarni Steig, Pál Weihe

**Affiliations:** University of the Faroe Islands, Tórshavn, Faroe Islands (M.S. Petersen, M. Strøm, E.H. Eliasen, M. Johansen, A.S. Veyhe, M.F. Kristiansen, S. Gaini, P. Weihe);; The Faroese Hospital System, Tórshavn (M.S. Petersen, E.H. Eliasen, M. Johansen, A.S. Veyhe, P. Weihe);; Faroese Food and Veterinary Authority, Tórshavn, (D.H. Christiansen, J.P. Fjallsbak);; National Hospital of the Faroe Islands, Tórshavn, (M.F. Kristiansen, S. Gaini, B. Steig);; COVID-19 Task Force, Ministry of Health, Tórshavn (M.F. Kristiansen, B. Steig);; Odense University Hospital, Odense, Denmark (S. Gaini);; University of Southern Denmark, Odense (S. Gaini);; Chief Medical Officer Office, Tórshavn (L.F. Møller)

**Keywords:** coronavirus disease, 2019 novel coronavirus disease, COVID-19, SARS-CoV-2, severe acute respiratory syndrome coronavirus 2, respiratory diseases, zoonoses, viruses, seroprevalence, antibodies, Faroe Islands, Denmark

## Abstract

We conducted a nationwide study of the prevalence of severe acute respiratory syndrome coronavirus 2 infection in the Faroe Islands. Of 1,075 randomly selected participants, 6 (0.6%) tested seropositive for antibodies to the virus. Adjustment for test sensitivity and specificity yielded a 0.7% prevalence. Our findings will help us evaluate our public health response.

The magnitude of the coronavirus disease (COVID-19) pandemic is unknown because of a relatively large proportion of presumably asymptomatic persons (*1*–*3*). Reported infection rates, which mostly rely on PCR-based testing of symptomatic persons, may underestimate underlying infection rates. Analysis of severe acute respiratory syndrome coronavirus 2 (SARS-CoV-2)–specific antibodies is required to more accurately guide COVID-19 response and calibrate public health efforts.

In the Faroe Islands, a geographic isolate of 52,154 inhabitants, the first COVID-19 case occurred on March 3, 2020. From early in the pandemic, the Faroe Islands adhered to the official recommendations by the World Health Organization of an active suppression strategy with high numbers of testing, contact tracing, and quarantine of infected persons and close contacts (M.F. Kristiansen et al., unpub. data). We aimed to estimate the population prevalence of SARS-CoV-2 infection by serotesting for antibodies in a nationwide sample of randomly selected inhabitants of the Faroe Islands (Appendix).

From the Faroese Population Registry, we randomly sampled 1,500 persons and invited them by letter to a clinical visit at 1 of 6 study sites around the islands mainly during week 18 (April 27–May 1, 2020) independently of previous positive PCR test result. To persons unable to attend a testing site, we offered home visits. Nonresponders received a follow-up phone call. We obtained informed consent from all participants; parents signed the consent form for their children <18 years of age. The Faroese Ethical Committee and the Data Protection Agency approved the study.

We conducted SARS-CoV-2–specific antibody (IgG, IgM) analyses on serum samples by using the commercial Wantai SARS-CoV-2 Ab ELISA kit (Beijing Wantai Biologic Pharmacy Enterprise, http://www.ystwt.cn), according to the manufacturer’s instructions. We estimated the 95% CI for crude prevalence using exact binomial models and for prevalence adjusted for test performance as reported by the producer (sensitivity (94.4% [95% CI 90.9–96.8]) and specificity (100% [95% CI 98.8–100.0]) using bootstrap methods (*4*). We used SPSS Statistics 25 (IBM, Inc., https://www.ibm.com) for the analysis.

Of 1,500 persons invited to the study, 1,141 (76.1%) provided consent and 1,075 (71.7%) were tested (Figure). Mean age of participants was 42.1 years (SD ± 23.1, range 0–100 years); 50% were women (Table). The study sample was representative of the entire population (Table) regarding geography, sex, and age; the representativeness of the youngest (0–9 years) participants and participants 60–69 years of age was slightly less. Nonparticipants were more often men and significantly younger than participants (32.6 [SD ± 26.7] vs. 42.9 years [SD ± 23.2]; p<0.01), and geographic distribution was comparable (p = 0.7).

**Figure:**
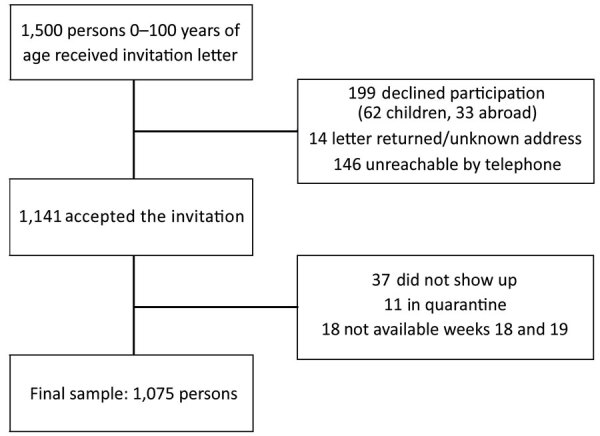
Study participation and reasons for dropout in a seroprevalence analysis of severe acute respiratory syndrome coronavirus 2–specific antibodies, Faroe Islands, 2020.

**Table:** Comparison of study participants and the entire population in a seroprevalence analysis of severe acute respiratory syndrome coronavirus 2–specific antibodies, Faroe Islands, April 27–May 1, 2020

Characteristic	No. (%)	Population distribution, no. (%)	No. seropositive	Crude prevalence for antibody % (exact binomial 95%CI)
Entire sample	1,075	52,154	6	0.6 (0.1–1.2)
Sex				
M	538 (50.2)	26,987 (51.7)	3	0.6 (0.1–1.6)
F	537 (49.8)	25,167 (48.3)	3	0.6 (0.1–1.6)
Age group, y				
0–9	92 (8.6)	7,164 (13.7)*	0	0
10–19	150 (14.0)	7,335 (14.1)	1	0.7 (0.0–3.7)
20–29	116 (10.8)	5,966 (11.4)	1	0.9 (0.0–4.7)
30–39	126 (11.7)	6,298 (12.1)	0	0
40–49	148 (13.8)	6,522 (12.5)	0	0
50–59	154 (14.3)	6,718 (12.9)	1	0.6 (0.0–3.6)
60–69	151 (14.0)	5,665 (10.9)*	1	0.7 (0.0–3.6)
70–79	98 (9.1)	4,201 (8.1)	2	2.0 (0.2–7.2)
80–89	30 (2.8)	1,848 (3.5)	0	0
>90	10 (0.9)	437 (0.8)	0	0
Geographic area				
Streymoy	530 (49.3)	24,926 (47.8)	3	0.6 (0.1–1.6)
Eysturoy	251 (23.3)	11,782 (22.6)	1	0.4 (0.0–2.2)
Norðoyggjar	114 (10.6)	6,206 (11.9)	0	0
Vágar	74 (6.9)	3,366 (6.5)	1	1.4 (0.0–7.3)
Sandoy og Suðuroy	106 (10.0)	5,874 (11.2)	1	0.9 (0.0–5.1)

*Significant difference, p<0.05.

Six persons (3 women, 3 men) tested positive for SARS-CoV-2–specific antibodies (0.6% [exact binomial 95% CI 0.2%–1.2%]). One of the 6 positive persons had previously confirmed infection by PCR; the others had not been tested, although 2 reported symptoms. After adjustment for test sensitivity and specificity, the prevalence of SARS-CoV–2-specific antibodies was 0.7% (bootstrap 95% CI 0.3%–1.3%).

The crude seroprevalence of SARS-CoV-2 antibodies (0.6% [adjusted 0.7%]) in our randomly selected population-based sample corresponds to 313 SARS-CoV-2–seropositive persons in the population, which is somewhat higher than the number of confirmed infections (187 cases [crude prevalence 0.4%]) in the Faroe Islands on June 6 (*5*). The number of active COVID-19 cases peaked on March 23 when the prevalence was 196 cases/100,000 persons, and the last locally transmitted case was diagnosed April 22. The low number of undetected cases found in this study supports the effectiveness of the extensive testing regime, contact tracing, and quarantining in mitigating the virus. The exact seroprevalence levels from the few published studies included in a recent meta-analysis are highly region-dependent; levels ranged from 2.8% to 31.5% (J. Levesque, D.W. Maybury, unpub data, https://doi.org/10.1101/2020.05.03.20089201). Contrary to the other studies, the participants in our study sample were unselected and representative of the background population with respect to age, sex, and geographic area, making selection bias an unlikely explanation of our results.

Major strengths of our study include the high participation rate and the representativeness of the randomly selected study sample, although the youngest children were slightly underrepresented. Rather than a flow immunoassay test, we used the ELISA that performed best of 9 commercial SARS-CoV-2 immunoassays (R. Lassaunière et al., unpub. data, https://doi.org/10.1101/2020.04.09.20056325). However, we acknowledge that our estimates could change with new information about test accuracy of kits used, and cross-reactivity with other infections might be a challenge in antibody testing, but evidence on serologic testing is limited. Although antibodies might be undetected during early stages of the disease (*6*), our sample collection occurred 5–10 days after the last case in the Faroe Islands was detected, which makes this possibility unlikely in explaining the low proportion tested seropositive.

Our findings will help us evaluate the effect of public health efforts in the Faroe Islands. In addition, our findings will help guide the COVID-19 response moving forward, ensuring the previously held belief that few undetected cases were present in the Faroe Islands.

Appendix Descriptive epidemiology of coronavirus disease, Faroe Islands.
