# Supplementary material for: Seroprevalence of SARS-CoV-2–Specific Antibodies, Faroe Islands
Source: Emerg Infect Dis. 2020 Nov;26(11):2760–2. doi: 10.3201/eid2611.202736 (PMC7588539; doi:10.3201/eid2611.202736)
Supplement: Appendix — Descriptive epidemiology of coronavirus disease, Faroe Islands. [file 20-2736-Techapp-s1.pdf]

Article DOI: https://doi.org/10.3201/eid2611.202736

Seroprevalence of SARS-CoV-2–Specific Antibodies, Faroe Islands, Denmark

Appendix

Short Information Regarding Descriptive Epidemiology of COVID-19 in Faroe Islands

The Faroe Islands, a small geographically isolated island population situated between Iceland, Norway, and Scotland with 52,428 inhabitants, had its first confirmed case of COVID-19 on March 3, 2020, and the last confirmed case diagnosed on April 22 and recovered on May 8, i.e., COVID-19 is currently eliminated. In total, 187 cases have tested positive, corresponding to 357 per 100,000 which as of May 8 was the 12th highest confirmed cases per capita (https://www.worldometers.info/coronavirus/#countrie).

The strategy of the Faroe Islands followed the recommendations of the World Health Organization (WHO), with an active suppression strategy. The initial exponential growth diminished during the weeks after lockdown, etc., to a slow linear growth during early and mid-April and subsequently elimination. Of note, the Faroe Islands tested at a very high frequency, with the number of tests now equaling 27% of the population, and on May 8 when the last case recovered the Faroe Islands had the highest number of tests conducted per capita in the world (https://corona.fo). This large test capacity was due to a fast adaptation of the Faroese Food and Veterinary Authority (https://www.hfs.fo) to accommodate diagnostic real-time PCR resources normally used in salmon farming to test for COVID-19. Patients, including those with mild symptoms, are referred by medical doctors and general practitioners to drive-in testing facilities at all 3 Faroese hospitals. The threshold for testing was initially high the first days of the Faroese COVID-19 epidemic, but quickly the Faroe Islands lowered the testing threshold to comply with the WHO recommendations. Thus, the Faroe Islands have been very successful with their strategy in dealing with the COVID-19 epidemic and is the first in the Western Hemisphere to have eliminated the disease. The successful strategy was following the suggestions by the WHO with 1) maximum testing for COVID-19, 2) isolation/quarantine of COVID-19–positive persons and their close contacts, and 3) society lockdown/social distancing.

Of the 187 positive cases, 53% were in women and mean age was 40 years ranging from 0 to 92 years. The most common symptoms among the 187 cases were fever, headache, and cough, while 11.2% were asymptomatic. The proportion of asymptomatic cases seemed to vary by age, with 25 and 30% asymptomatic cases in the age groups 0–17 and $\geq$65 years, respectively, and only 6% asymptomatic cases in the age group 18–64 years. However, numbers of asymptomatic cases are small (n = 21 cases reported asymptomatic in total), and therefore should be interpreted with caution. Eight patients were admitted to hospital, but there were no fatalities or admissions to intensive care unit (M.F. Kristiansen et al, unpub. data).
